# Diarrhoea: An Inflammatory Disorder Mimicking an Infection

**DOI:** 10.7759/cureus.44101

**Published:** 2023-08-25

**Authors:** Usman Khan, Amr Elseby, Hassan Qureshi

**Affiliations:** 1 Internal Medicine, Royal Preston Hospital, Preston, GBR; 2 Family Medicine, Blackpool Victoria Hospital, Blackpool, GBR; 3 Internal Medicine, Blackpool Victoria Hospital, Blackpool, GBR

**Keywords:** inflammatory, infective, auto immune, behcet's disease, abdominal pain, diarrhoea, bloody stool

## Abstract

Diarrhoea is a common presentation in acute emergency with potentially multifactorial causes. Hence, the chances of misdiagnosis can be high. Among these causes are listed diseases which are primarily inflammatory in nature. Presented here is a rare case of Behcet’s colitis with valuable clinical lessons, in a patient who is known to have Behcet’s disease and was admitted with acute onset bloody diarrhoea. This was a gastrointestinal manifestation of the patient's multisystemic inflammatory disorder as proven by investigations via computed tomography (CT) scan, sigmoidoscopy, and histology. He was treated with steroids and mesalazine, and later switched to infliximab and made a quick recovery.

## Introduction

Behcet’s disease (BD) is a rare multisystem inflammatory condition characterized by recurrent oral ulcers and any of the other systemic manifestations, including skin and genital lesions, arthritis, uveitis, thrombophlebitis, mucocutaneous, or neurological involvement [1]. Its clinical features are commonly attributed to vasculitis, which involves blood vessels of all sizes but has an inclination towards venous circulation. Gastrointestinal complications account for 3-25% of the presentations of this inflammatory disorder, which can be similar to inflammatory bowel disease (IBD). It is interestingly noted that gastrointestinal symptoms are not part of the International Criteria for Behçet's Disease (ICBD) criteria as it is not sensitive [2]. This can make it difficult to distinguish and manage [3]. The etiology of this disease is unknown. The pathogenesis can be triggered by an infectious cause in a patient with a genetic predisposition combined with various environmental and external factors [1]. 

## Case presentation

A 37-year-old male was admitted with a three-day history of abdominal pain and mixed watery and bloody diarrhoea over the past 24 hours with bowel motion up to 20 times a day. The second presenting complaint was of severe oral ulceration limiting speech and oral intake. His medical history included a bone marrow transplant in 1991 due to acute lymphocytic leukaemia. Later in his life, he had Behcet’s oral involvement in 2020 (when he was found to be human leukocyte antigen (HLA) B27 positive) and was put on azathioprine (as maintenance therapy). He presented twice previously due to oral Behcet’s flare-up whilst on azathioprine.

On examination in the current presentation, he was found to be pyrexial with a temperature of 39 degrees Celsius and was mainly tender in the left iliac fossa. During the initial assessment, he was acutely unwell with a National Early Warning Score (NEWS) of 5. A digital rectal examination (DRE) revealed bloody-stained stool and he was clinically dry with multiple aphthous ulcers in his mouth on oral examination. Initial blood test results are shown in Table 1. 

**Table 1:** Significant laboratory results

Blood test results	Patient value	Reference range
C-reactive protein (CRP) (mg/L)	334	<5
Lactate (mmol/L)	4	0.5-2.2
Platelets (x109 /L)	80	140-350
Haemoglobin (g/L)	103	130-170
White cell count (WCC) (x109 /L)	2.3	4-11

**Figure 1:**
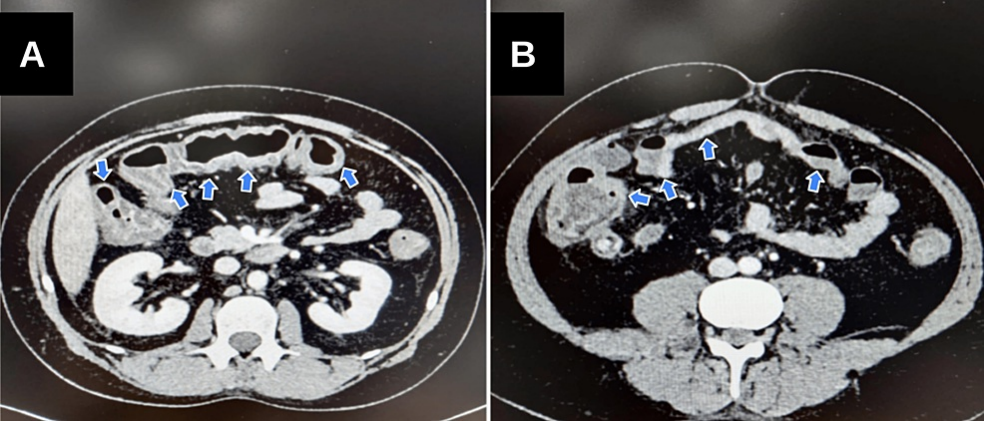
Transverse section of a contrast CT abdomen showing pancolitis with inflammatory stranding of pericolic mesentery in left colon and mucosal oedema in right colon (arrows)

He was subsequently treated as having sepsis secondary to intra-abdominal source due to infectious diarrhoea and started on amoxicillin and metronidazole as per trust guidelines and managed with intravenous (IV) fluids while azathioprine was kept on hold. Multiple stool cultures were taken including *Clostridium difficile*, *Campylobacter*, *Escherichia coli*, *Salmonella*, *Giardia*, *Shigella,* and *Cryptosporidium* and had three sets of blood cultures taken, all of which were negative. Computed tomography (CT) abdomen was done and showed pancolitis (Figure 1). 

**Figure 2:**
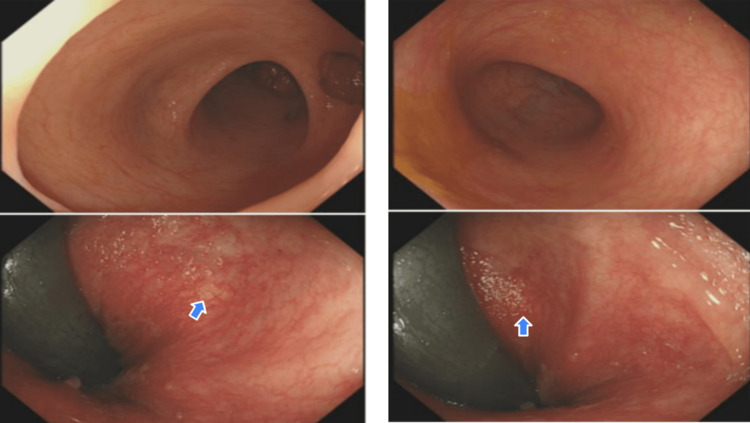
Sigmoidoscopy showing presence of discrete aphthous ulceration (arrows) in proximal descending, distal sigmoid colon, and rectum, an appearance suggestive of Behcet’s disease

Since the problem was thought of as primarily infectious in nature, the patient was admitted under the infectious disease team. Despite a seven-day treatment with IV antibiotics, there was minimal improvement in clinical condition and blood results. The patient deteriorated further thereby developing worsening thrombocytopenia with platelets of 16 x10^9^/L for which he had a unit of platelets. He was started on a trial of steroids on the gastroenterology team's advice and underwent a sigmoidoscopy (Figure 2). 

 A sample was sent for histology. Despite a weaning course of steroids, there was only some improvement in diarrheal frequency although the C-reactive protein (CRP) did improve to 114 mg/L. In view of inadequate improvement, mesalazine 4 g per day was added on day eight to his treatment after involving the rheumatology team. Within four days, there was a rapid improvement of his symptoms including healing of his oral ulcers. As a result, he was now able to speak fully and his diarrhoea and abdominal pain were resolved. He was discharged and later put on infliximab with a starting dose of 5 mg/kg, which further improved his condition. The biopsies taken later revealed findings inconsistent with IBD and in keeping with Behcet’s colitis. 

Histology later revealed ulceration with moderate active inflammation with no crypt abscess, distortion, or granulomas. This was suggestive of Behcet’s colitis. 

## Discussion

BD is a chronic multisystem immune-mediated inflammatory disease characterized by mucocutaneous involvement, relapsing oral and genital ulcers, ocular lesions, and vascular inflammation. It can cause intestinal problems and manifest as Behcet’s colitis in 3-25% of patients with BD and presents as abdominal pain, diarrhoea which is often bloody due to ulcers in the colon, and systemic symptoms of fever and weight loss [3].

The aetiology of this disease may represent aberrant immune activity triggered by exposure to infectious or environmental agents in patients with underlying genetic predisposition, especially those with HLAB51 and B27 genes [4]. It therefore shares many characteristics with IBD including genetic background, clinical manifestations, and therapeutic strategies [5]. Mucosal ulceration in BD tends to commonly affect the ileum, followed by the caecum and then other parts of the colon with a higher tendency of perforation with ileocecal ulcers [6]. This is similar to Crohn's disease (CD); however, ulceration tends to be more severe in CD. In addition, patients with gastrointestinal manifestations of BD will not have scalloping, ulceronodular patterns, and complications such as abscess and fistulae formation (which can be found in CD patients) [7]. 

Unfortunately, there isn't a pathognomic test for BD and the diagnosis is made based on the presence of clinical symptoms. The ICBD published a scoring system on the diagnosis of BD (if the score is ≥4). This scoring system includes ocular lesions (2 points), genital aphthosis (2 points), oral aphthosis (2 points), skin lesions (1 point), neurological manifestation (1 point), vascular manifestations (1 point), and positive pathergy test (1 point, this is optional but if done can add a point when found to be positive) [4]. Similarly, there isn't a formal diagnostic criteria for Behcet's Colitis. As a result, in this case, diagnosis was made based on the clinical presentation along with confirmation of discrete deep ulcers with defined edges, especially in the ileo-caecal region on endoscopy and based on histological findings of deep ulcers with non-specific inflammation. Sulfasalazine and steroids are the primary therapies used in intestinal BD although some steroid-sparing agents such as azathioprine also have a role. Infliximab (tumour necrosis factor (TNF)-α monoclonal antibody) has been found beneficial in patients not responsive to conventional therapies [3]. Despite research on this relatively rare clinical presentation of BD, there are no definitive guidelines to manage this condition [8]. 

## Conclusions

This was a case of a relatively uncommon presentation of a rare disease. It emphasizes the importance of exploring all probable causes of a relatively common presentation (diarrhoea). As BD is a relatively rare condition to encounter, it is important to look through the past medical history as missing it can lead to a potentially life-threatening condition and cause serious of gastrointestinal problems such as acute gastrointestinal haemorrhage, obstruction, and perforation. This case also highlights the importance of imaging used, especially when there is no response to treatment or in patients who are acutely unwell. Moreover, inflammatory disorder flare-ups can present in the form of a clinical picture of sepsis and therefore should be considered as strong differentials. Rapid detection of this and early treatment with steroids and steroid-sparing agents can lead to better prognosis in patients with BD.
